# Development of a risk scoring system for evaluating the prognosis of patients with Her2-positive breast cancer

**DOI:** 10.1186/s12935-020-01175-1

**Published:** 2020-04-15

**Authors:** Chundi Gao, Jing Zhuang, Huayao Li, Cun Liu, Chao Zhou, Lijuan Liu, Fubin Feng, Changgang Sun, Jibiao Wu

**Affiliations:** 1 College of First Clinical Medicine, Shandong University of Traditional Chinese Medicine, Jinan, 250014 Shandong People’s Republic of China; 2 College of Basic Medical, Shandong University of Traditional Chinese Medicine, Jinan, 250014 Shandong People’s Republic of China; 3 Departmen of Oncology, Weifang Traditional Chinese Hospital, Weifang, 261041 Shandong People’s Republic of China; 4 Cancer and Immunology Institute, Shandong University of Traditional Chinese Medicine, Jinan, Shandong People’s Republic of China

**Keywords:** Her2-positive breast cancer, Prognostic risk scoring system, Lasso Cox regression analysis, Copy number variation, Survival analysis

## Abstract

**Background:**

As one of the many breast cancer subtypes, human epidermal growth factor receptor 2 (Her2)-positive breast cancer has higher invasiveness and poor prognosis, although the advent of anti-Her2 drugs has brought good news to patients. However, the emergence of drug resistance still limits its clinical efficacy, so there is an urgent need to explore new targets and develop a risk scoring system to improve treatments and evaluate patient prognosis.

**Methods:**

Differentially expressed mRNAs associated with Her2-positive breast cancer were screened from a TCGA cohort. The prognostic risk scoring system was constructed according to univariate and Lasso Cox regression model analyses and combined with clinical factors (such as age and TNM) for univariate and multivariate analyses to verify the specificity and sensitivity of the risk scoring system. Finally, based on correlation and CNV mutation analyses, we explored the research value of the mRNAs involved in the system as key genes of the model.

**Results:**

In this study, six mRNAs were screened and identified to construct a prognostic risk scoring system, including four up-regulated mRNA (RDH16, SPC25, SPC24, and SCUBE3) and two down-regulated mRNA (DGAT2 and CCDC69). The risk scoring system can divide Her2-positive breast cancer samples into high-risk and low-risk groups to evaluate patient prognosis. In addition, whether through the time-dependent receiver operating characteristics curve or compared with clinical factors, the risk scoring system showed high predictive sensitivity and specificity. Moreover, some CNV mutations in mRNA increase patient risk by influencing expression levels.

**Conclusion:**

The risk scoring system constructed in this study is helpful to improve the screening of high-risk patients with Her2-positive breast cancer and is beneficial for implementing early diagnosis and personalized treatment. It is suggested that these mRNAs may play an important role in the progression of Her2-positive breast cancer.

## Background

Breast cancer is a common malignant tumor that poses a serious threat to women. Although the level of comprehensive treatment is increasing, breast cancer incidence and recurrence rates continue to increase yearly. Human epidermal growth factor receptor 2 (Her2)-positive breast cancer, which is one of many breast cancer subtypes, has attracted much attention because of its strong invasiveness, poor tumor-free survival, and poor overall survival (OS) rate [1–3]. Presently, with the advent of numerous anti-Her2 molecular-targeted therapies, the treatment strategy and prognosis of Her2-positive breast cancer patients have been greatly improved. However, drug resistance caused only by the restriction of the Her2 gene is inevitable. Therefore, evaluating and improving the prognosis of patients with Her2-positive breast cancer remains a challenging and daunting task. There is an urgent need to further develop new specific biomarkers and build stable independent risk prediction models to improve treatment and evaluate patient prognosis, which has an important impact on clinical decision-making and patient counseling [4].

As the mechanism of tumorigenesis continues to be explored, effective biomarkers with diagnostic and prognostic significance have been revealed. Gene copy number variation (CNV) caused by genomic instability has received attention as a potential parameter for the diagnosis of some tumor subtypes [5]. Among these, it has been found that the CNV-based classifier constructed by comparing the CNV pattern of lung adenocarcinoma and lung squamous cell carcinoma can be used to distinguish two subgroups of non-small cell lung cancer [6]. Xu et al. found that CNV analysis could help to identify patients with chronic liver disease who are at high risk of hepatocellular carcinoma (HCC), which is of great value for early monitoring and intervention of HCC. In addition, Studies by Pan et al. have shown that the CNVs of PGAP3, GRB7, MIR4728, PNMT, STARD3, TCAP, and ERBB2 play an important role in the accurate diagnosis of breast cancer subtypes, which further reveals the differences between these subtypes and improves the accuracy of breast cancer diagnosis [7]. Thus, tumor-specific CNVs can be used as a new detection tool for early diagnosis and treatment intervention of tumors.

In recent years, based on the integrated analysis of high-throughput data in the TCGA database, mRNA, microRNA, and DNA methylation models have been proposed as predictors of cancer progression risk [8–10]. Furthermore, based on the integrated analysis of high-throughput data in the TCGA database, mRNA, microRNA, and DNA methylation models have been recently proposed as predictors of cancer progression risk, and some progress has been made in constructing prognostic risk models and evaluating the prognosis of cancer patients.

## Methods

### Data source

Data regarding mRNA expression, CNVs, and clinical information related to Her2-positive breast cancer were obtained from The Cancer Genome Atlas database (TCGA: https://cancergenome.nih.gov/) [11], including 22 normal samples and 161 Her2-positive breast cancer samples. In total, 183 samples were included in the study; Her2-positive breast cancer samples with incomplete prognostic information were excluded. Finally, a total of 156 Her2-positive breast cancer samples were selected for further construction of an mRNA-related prognostic risk model. TCGA was used as a public open database, and the relevant information retrieved from it did not require further ethical approval.

### Differential expression and pathway enrichment analysis

Based on the edger software package, the normal and Her2-positive breast cancer expression data downloaded from the TCGA database were standardized, and the differences were analyzed to obtain abnormal mRNA expression. In addition, in order to understand the functional abnormalities caused by abnormal mRNA expression in patient activities and to explore the possible biological processes and potential pathways, the David database for annotation, visualization, and comprehensive discovery (http://david.abcc.ncifcrf.gov/) was used to perform functional enrichment analysis between mRNA [12], and P < 0.05 was used as the cut-off condition for screening Gene Ontology (GO) and Kyoto Encyclopedia of Genes and Genomes (KEGG) pathway enrichment analysis.

### Construction of the prognostic risk scoring system for Her2-positive breast cancer

In order to construct the mRNA prognostic risk scoring system for Her2-positive breast cancer, univariate Cox regression analysis was used to determine the prognostic gene. P < 0.01 gene was considered as significant for positive Her2 prognosis. Then, Lasso penalized Cox regression analysis was used to further select OS-related prognostic genes in Her2-positive breast cancer patients. Least absolute shrinkage and selection operator (LASSO) is a generalized linear regression algorithm [13] that can select and regularize variables simultaneously, because the complexity of Lasso regression is controlled by the coefficient lambda. Therefore, the model with fewer variables can be obtained by executing the penalty ratio according to their size. Using the cross-validation program cv.glmnet to determine the lambda value with minimum error to further reduce the number of prognostic mRNAs selected by univariate COX analysis, the risk scoring system can be constructed.

To further determine the survival risk, a risk scoring system was constructed based on Her2-positive breast cancer mRNA data using the following formula:

$${\text{Risk score}} = \sum\limits_{i = 1}^{N} {Exp_{i} \times \beta_{i} }$$

Here, β represents the mRNA coefficient and Exp represents the mRNA expression value. Using the median risk score as the critical value, the patients with Her2-positive breast cancer in the TCGA database were divided into low-risk and high-risk groups. The survival rate of each group was compared by Kaplan–Meier (Km) and log-rank methods. The time-dependent receiver operating characteristic (ROC) curve was drawn using the R “timeROC” software package to evaluate the specificity and sensitivity of the risk scoring system based on mRNA expression. P < 0.05 showed statistical significance.

### Predictive ability of the risk scoring system combined with clinical factors

In this study, the clinical data of Her2-positive breast cancer patients obtained from the TCGA database included age, stage, TNM stage, survival time, and survival status. In order to further test the prognostic performance of the risk scoring system and evaluate whether it can perform independently from other clinical parameters (including age, stage, and TNM stage), the Cox regression model was used for univariate and multivariate analyses. P < 0.05 was considered as statistically significant.

### Correlation of mRNA and CNV mutation analysis in the risk scoring system

In order to explore differences and correlations between the mRNA in normal and Her2-positive breast cancer samples in the risk scoring system, We not only conducted correlation analysis, but also showed the CNV mutation of mRNA in the scoring system by combining with CNV data, analyzed CNV mutation and mRNA expression, and identified a relationship between mutation and expression. The purpose of this study is to provide favorable theoretical support for the early diagnostic and prognostic evaluation of Her2-positive breast cancer.

## Results

### Identification of differentially expressed mRNAs

**Fig. 1:**
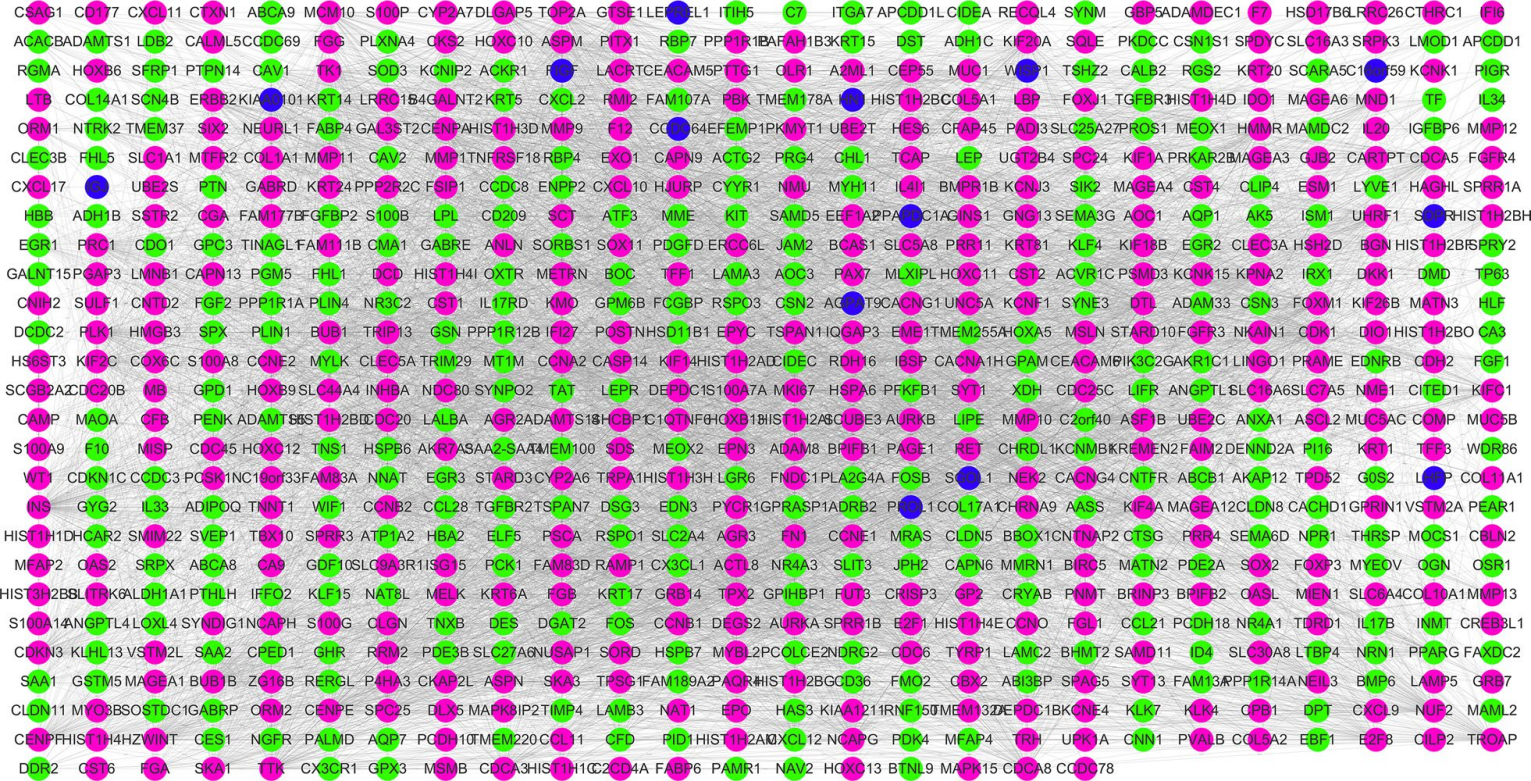
The PPI network of differentially expressed mRNAs in HER2-positive breast cancer, purple represents up-regulated mRNAs, green represents down-regulated mRNAs, and blue represents excavated related mRNAs

In this study, we downloaded an mRNA expression dataset of 183 samples and calculated mRNA expression between 22 normal samples and 161 Her2-positive breast cancer samples. The Edger software package was used to extract and analyze the differentially expressed data. Using | LogFC | ≥ 2 and P < 0.05 as filter cutoff criteria, 761 differentially expressed mRNAs were obtained, including 432 up-regulated mRNA and 329 down-regulated mRNA (Fig. 1).

### Enrichment analysis of GO functions and KEGG pathways

In order to understand the possible functional abnormalities caused by abnormal mRNA expression in patients, GO and KEGG analysis showed that these abnormally expressed mRNAs could be involved in many cancer-related pathways and biological processes. The enrichment results of functional and KEGG pathways showed that with p < 0.05 as the cutoff condition, these mRNA mainly involve 21 pathways (Table 1), including the PI3K-AKT, PPAR, and AMPK signaling pathways; cytokine–cytokine receptor interaction; cell cycle; and so on. GO analysis showed that differentially expressed mRNA was particularly abundant in the classification of molecular functions, biological processes, and cellular components (Table 2). As shown in Table 2, mRNA in the biological process (BP) group is mainly enriched in signal transduction, cell division, cell proliferation, negative regulation of apoptotic process, and so on. Molecular functional (MF) groups are mainly enriched in various combinations, such as protein binding, protein kinase binding and transcriptional activator activity, and RNA polymerase II core promoter proximal region sequence-specific binding. In addition, the cell component (CC) term is mainly related to the extracellular region and proteinaceous extracellular matrix. These most significant GO terms and KEGG pathways showed gene interactions at the functional level.

**Table 1:** KEGG pathway analysis about the differential mRNAs in HER2-positive breast cancer

Category	Term	Count	P value
KEGG_PATHWAY	hsa04151:PI3K-Akt signaling pathway	30	7.56E−04
KEGG_PATHWAY	hsa04060:Cytokine-cytokine receptor interaction	24	5.35E−04
KEGG_PATHWAY	hsa05034:Alcoholism	23	1.23E−05
KEGG_PATHWAY	hsa05203:Viral carcinogenesis	20	0.002068396
KEGG_PATHWAY	hsa04510:Focal adhesion	19	0.005049337
KEGG_PATHWAY	hsa04110:Cell cycle	18	3.46E−05
KEGG_PATHWAY	hsa05322:Systemic lupus erythematosus	18	9.40E 05
KEGG_PATHWAY	hsa04114:Oocyte meiosis	16	1.18E− 04
KEGG_PATHWAY	hsa03320:PPAR signaling pathway	14	6.67E−06
KEGG_PATHWAY	hsa04512:ECM-receptor interaction	14	1.20E−04
KEGG_PATHWAY	hsa04152:AMPK signaling pathway	14	0.003364429
KEGG_PATHWAY	hsa04914:Progesterone-mediated oocyte maturation	11	0.005364163
KEGG_PATHWAY	hsa04974:Protein digestion and absorption	11	0.005818118
KEGG_PATHWAY	hsa04610:Complement and coagulation cascades	10	0.003486826
KEGG_PATHWAY	hsa04923:Regulation of lipolysis in adipocytes	9	0.00327098

^a^If there were more than five terms enriched in this category, top 15 terms were selected according to count value. Count: the number of enriched mRNAs in each term

**Table 2:** Gene ontology analysis of mRNAs associated with HER2-positive breast cancer

Category	Term	Count	P value
GOTERM_BP_DIRECT	GO:0007165 ~ signal transduction	61	0.028781941
	GO:0045944 ~ positive regulation of transcription from RNA polymerase II promoter	55	0.012328036
	GO:0051301 ~ cell division	43	2.54E−10
	GO:0008284 ~ positive regulation of cell proliferation	43	9.78E−07
	GO:0000122 ~ negative regulation of transcription from RNA polymerase II promoter	41	0.025358743
GOTERM_CC_DIRECT	GO:0070062 ~ extracellular exosome	168	2.12E−08
	GO:0005576 ~ extracellular region	160	2.27E−28
	GO:0005615 ~ extracellular space	147	3.61E−30
	GO:0005578 ~ proteinaceous extracellular matrix	43	2.09E−14
	GO:0031012 ~ extracellular matrix	39	1.74E−10
GOTERM_MF_DIRECT	GO:0005515 ~ protein binding	379	0.003363112
	GO:0005509 ~ calcium ion binding	61	2.17E−08
	GO:0042802 ~ identical protein binding	43	0.012433807
	GO:0042803 ~ protein homodimerization activity	42	0.01295918
	GO:0046982 ~ protein heterodimerization activity	35	3.60E−04

^a^If there were more than five terms enriched in this category, top five terms were selected according to count value

### Derivation of the Her2-positive breast cancer risk scoring system

**Fig. 2:**
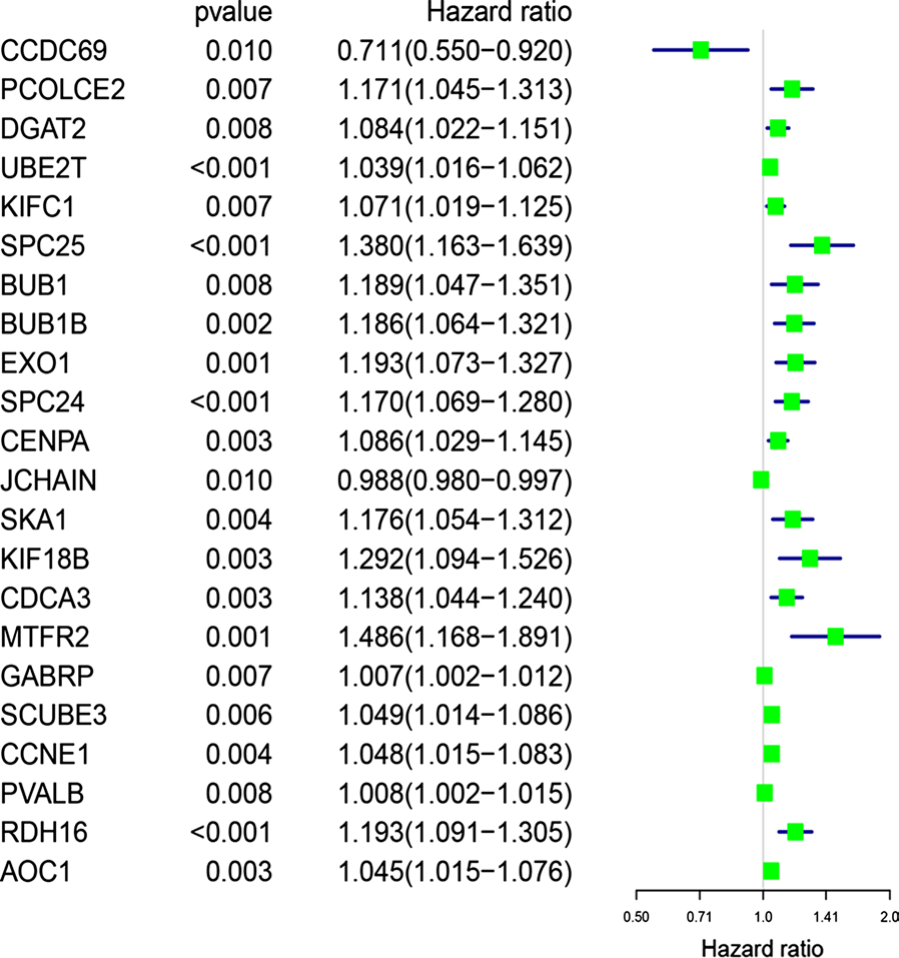
22 mRNAs associated with survival in Her2-positive breast cancer patients

**Fig. 3:**
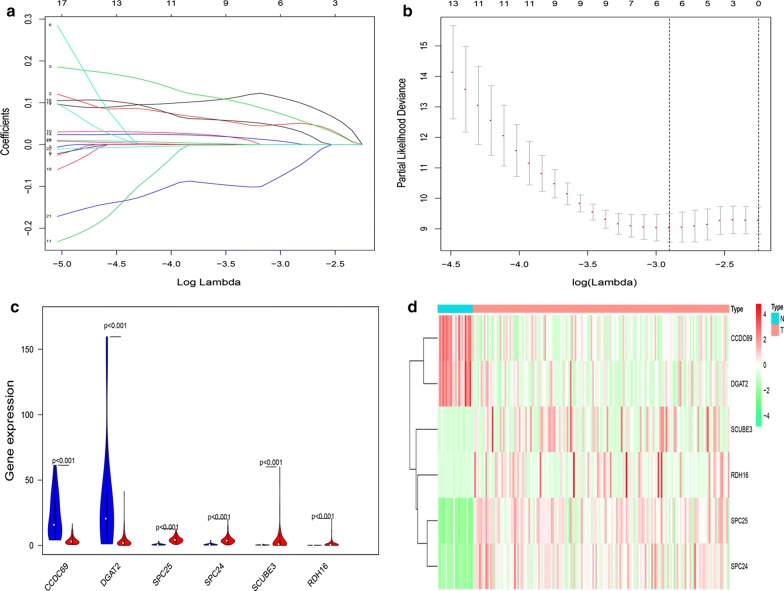
6 mRNAs were identified for construction of the risk scoring system. **a** Validation was performed for tuning parameter selection through the LASSO regression model; **b** Elucidation for LASSO coefficient profiles of prognosis-related mRNAs; **c** the expression of 6 mRNAs in the risk scoring system, red for HER2-positive breast cancer samples and blue for normal samples; **d** A heat map showing the expression of six mRNAs. The color from green to red shows a trend from low expression to high expression

According to the results of univariate Cox regression analysis, 22 survival-related mRNAs were initially screened for the construction of the Her2-positive breast cancer risk scoring system with P < 0.01 as the critical value (Fig. 2). Then, the lambda value with minimum error was determined by the cross-validation program, and six mRNAs with non-zero coefficients were defined to construct the final risk scoring system, namely RDH16, SPC25, SPC24, SCUBE3, DGAT2, and CCDC69 (Fig. 3). Based on the Lasso Cox regression model, the risk score was determined for each sample based on the status of the six mRNAs: Risk score = (0.106099941 × RDH16 expression) + (0.049775957 × SPC25 expression) + (0.065686112 × SPC24 expression) + (0.004864127 × SCUBE3 expression) + (0.037561146 × DGAT2 expression) + (− 0.061728085 × CCDC69 expression).

**Fig. 4:**
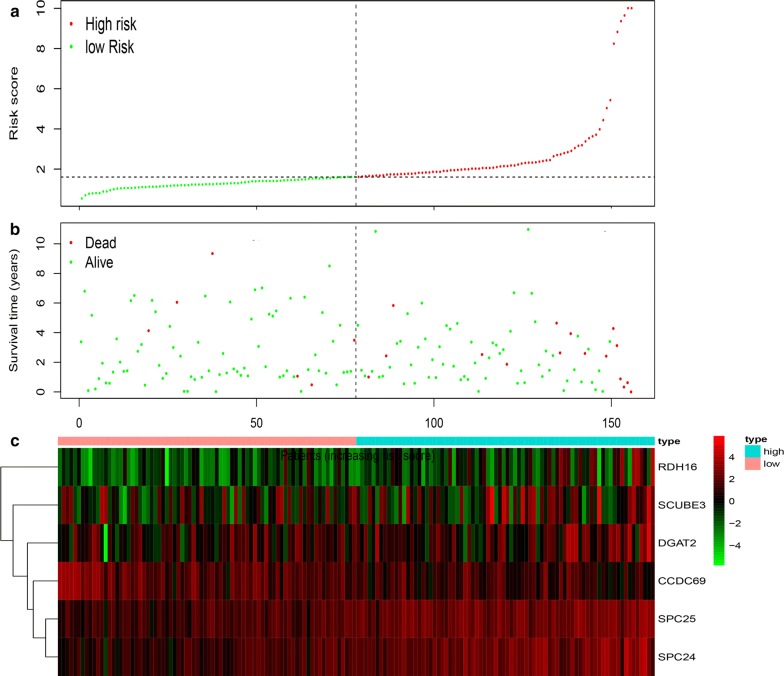
A risk scoring system was derived from the prognostic 6-mRNAs signature. **a** The risk score distribution on the basis of the 6-mRNAs signature; **b** the vital status of 156 patients with HER2-positive breast cancer in high- and low-risk groups; **c** Heatmap of the 6-mRNAs expression genomes in low- and high-risk levels

According to the median risk score as the critical value, 156 Her2-positive samples with prognostic information were divided into high-risk and low-risk groups, in which the OS in the low-risk group was significantly longer than that in the high-risk group (Fig. 4). In addition, the results showed that five of the six mRNAs defined by the Lasso Cox regression model (RDH16, SPC25, SPC24, SCUBE3, and DGAT2) showed positive coefficients, indicating that these mRNAs were closely related to the prognostic risk of Her2-positive breast cancer patients, and higher expression corresponded to shorter OS. However, CCDC69 showed a negative coefficient, indicating that the lower the expression, the shorter the OS of the patient.

### Analysis of the combination of the risk scoring system and clinical factors

**Fig. 5:**
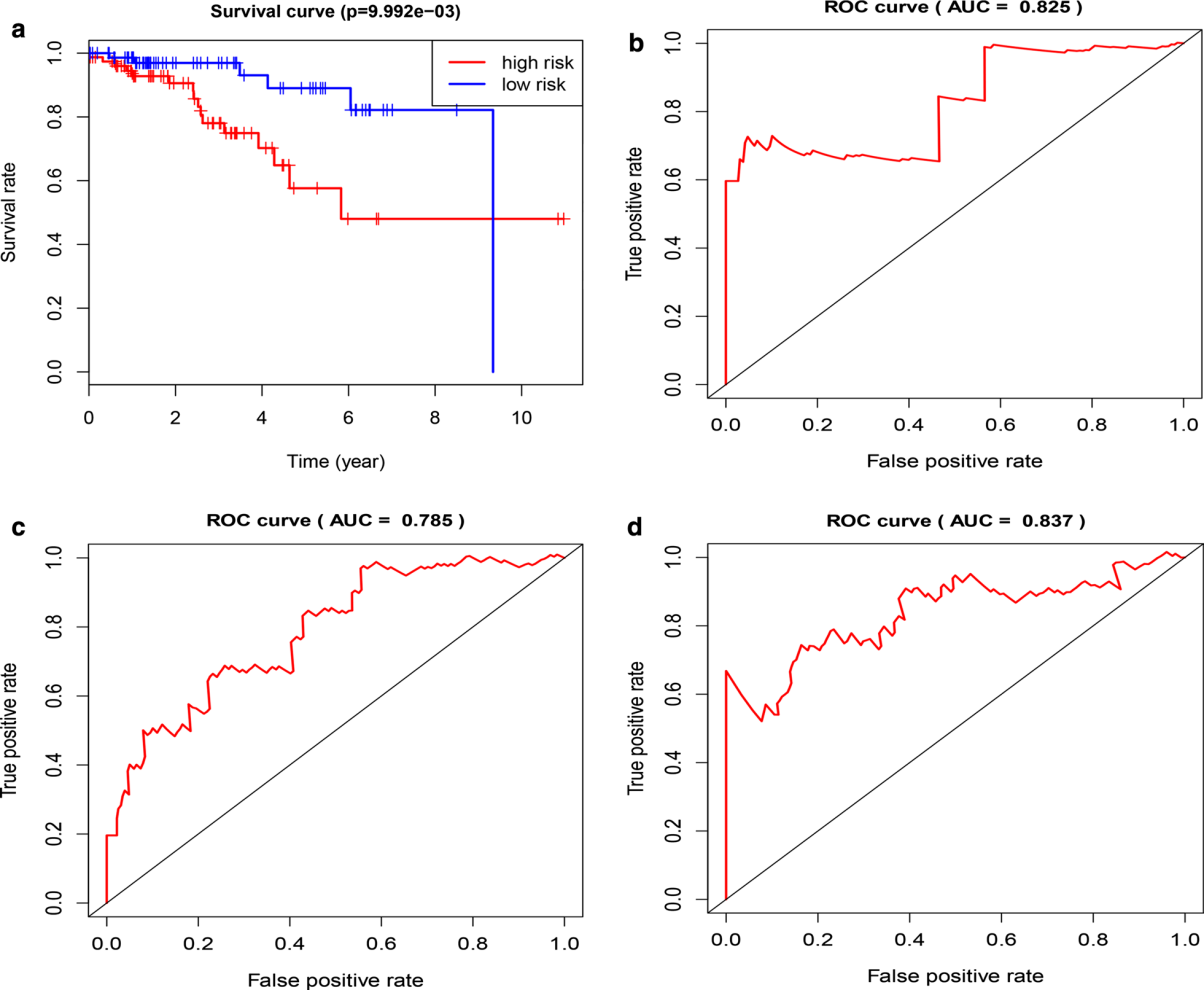
Kaplan–Meier and Time-dependent ROC curves for a risk scoring system based on 6 mRNAs. **a** Kaplan–Meier survival curves for overall survival. **b** ROC curves analysis for 1-,3-,5-year survival prediction

**Fig. 6:**
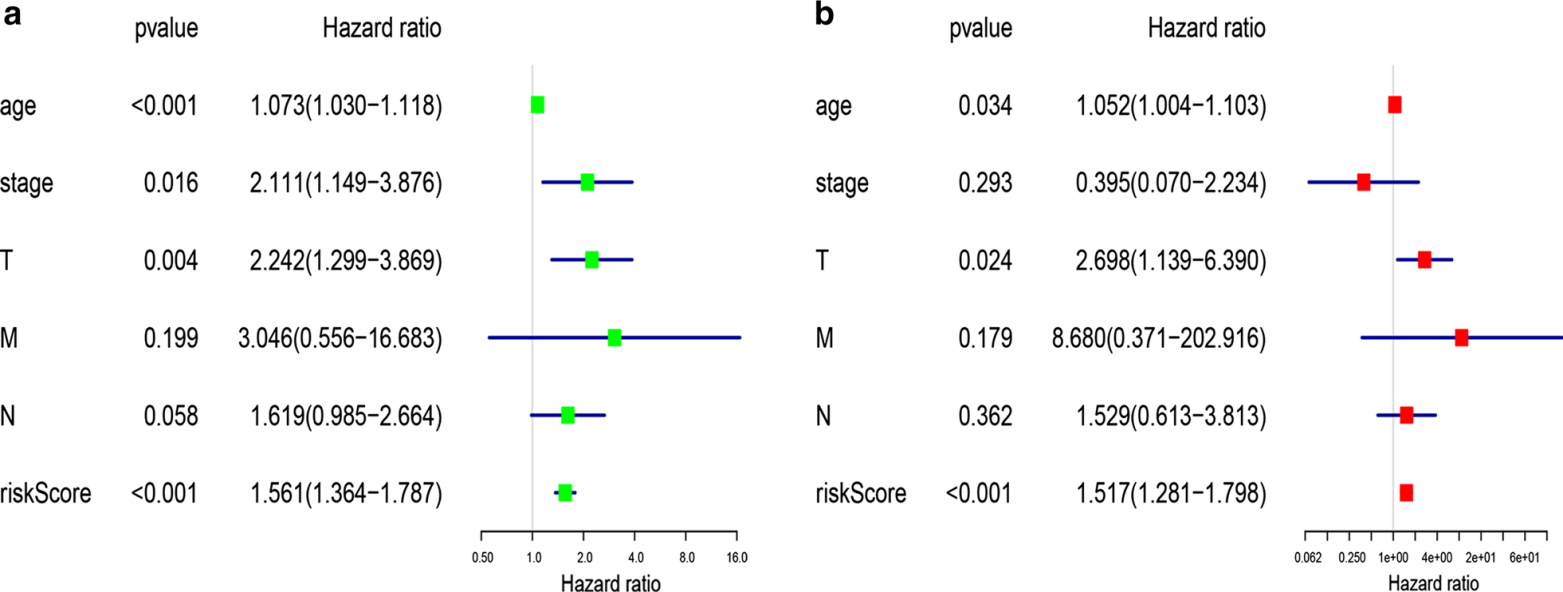
Univariate and multivariate analysis of HER2-positive breast cancer samples in TCGA database

According to the analysis of univariate and multivariate Lasso Cox regression models, we found that the risk scoring system constructed using six mRNAs could be used to predict the OS of Her2-positive patients. To further evaluate its predictive performance, we constructed the ROC curve and found that the predicted AUC values in 1, 3, and 5 years were 0.825, 0.785, and 0.837, respectively (Fig. 5). This means that the risk scoring system had excellent predictive power. In addition, we included clinical factors in this study (Table 3), and univariate Cox analysis showed that age, stage, T stage, risk score, and other clinical characteristics were closely related to OS with statistical significance. Further multivariate Cox analysis found that age, T stage, and risk score could be used as independent prognostic factors to evaluate the survival time of patients (Fig. 6). Regardless of univariate or multivariate analysis, the risk scoring system we constructed can evaluate prognosis very effectively, which further shows the evaluation value of the model.

**Table 3:** The specific baseline clinicopathological characteristics of 156 Her2-positive breast cancer samples

	156 Her2-positive breast cancer samples
Age	
< 60 years	75
≥ 60 years	81
Stage	
I	18
II	92
III	41
IV	3
Unknown	2
Pathologic T stage	
T1–2	133
T3–4	23
Pathologic N stage	
N0–1	119
N2–3	35
Unknown	2
Pathologic M stage	
M0	132
M1	3
Unknown	21
Survival time	
≤ 1 year	37
1 year ≤ 3 years	62
3 years ≤ 5 years	31
> 5 years	26

### An in-depth study of mRNA in the scoring system

**Fig. 7:**
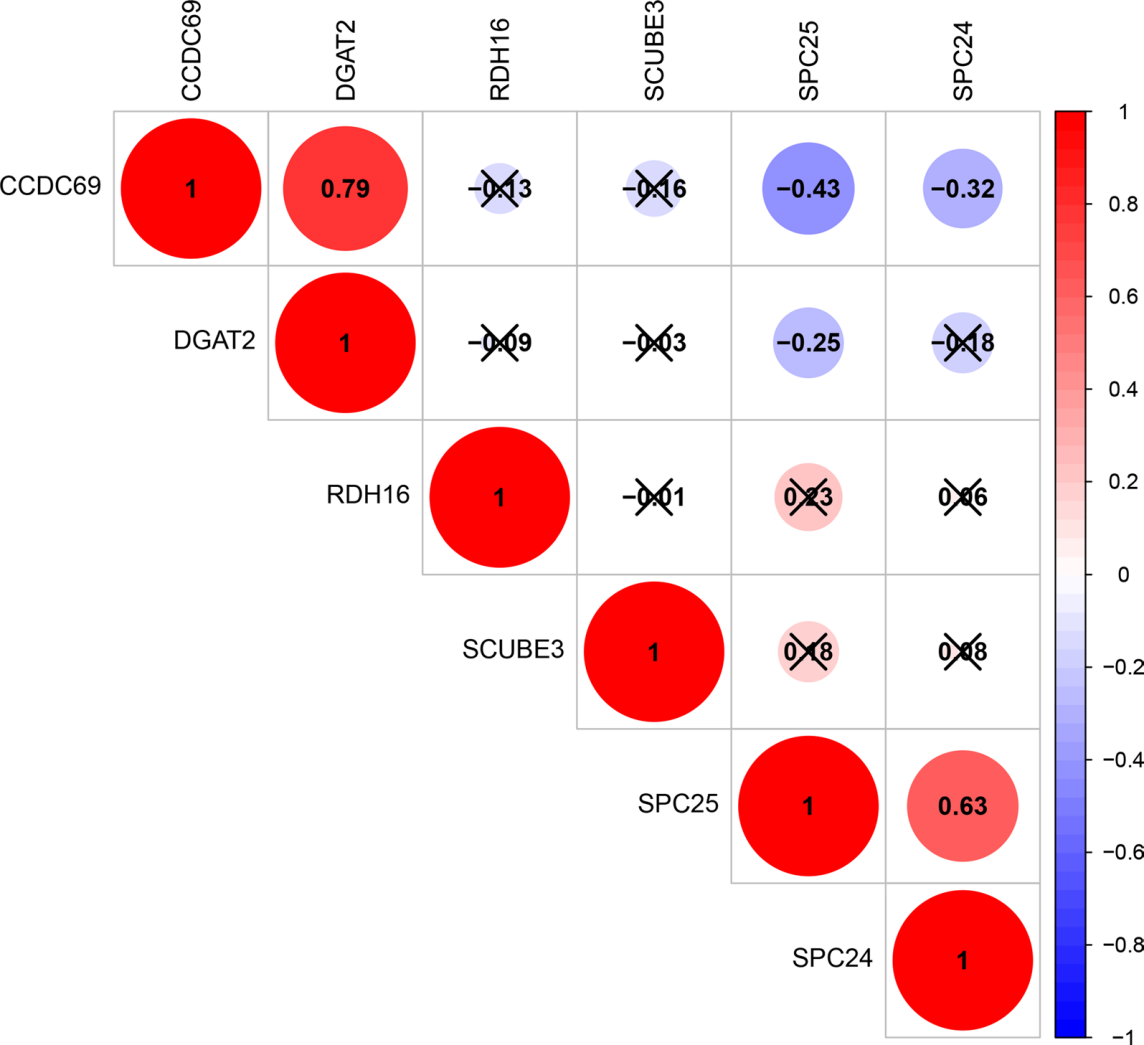
Correlation analysis of 6 mRNAs in the risk scoring system

**Fig. 8:**
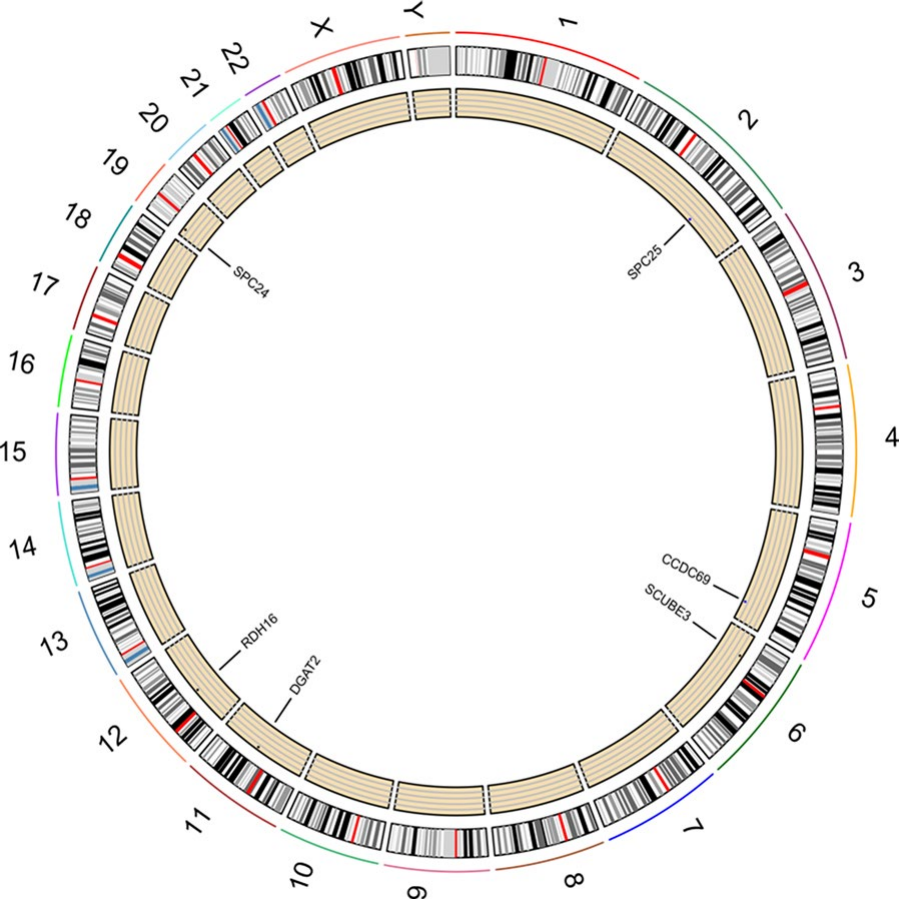
The CNV circle map of HER2-positive breast cancer-related mRNAs on chromosomes. The peripheral points indicate an increase in copy number, while the points in the inner indicate a decrease in copy number

**Fig. 9:**
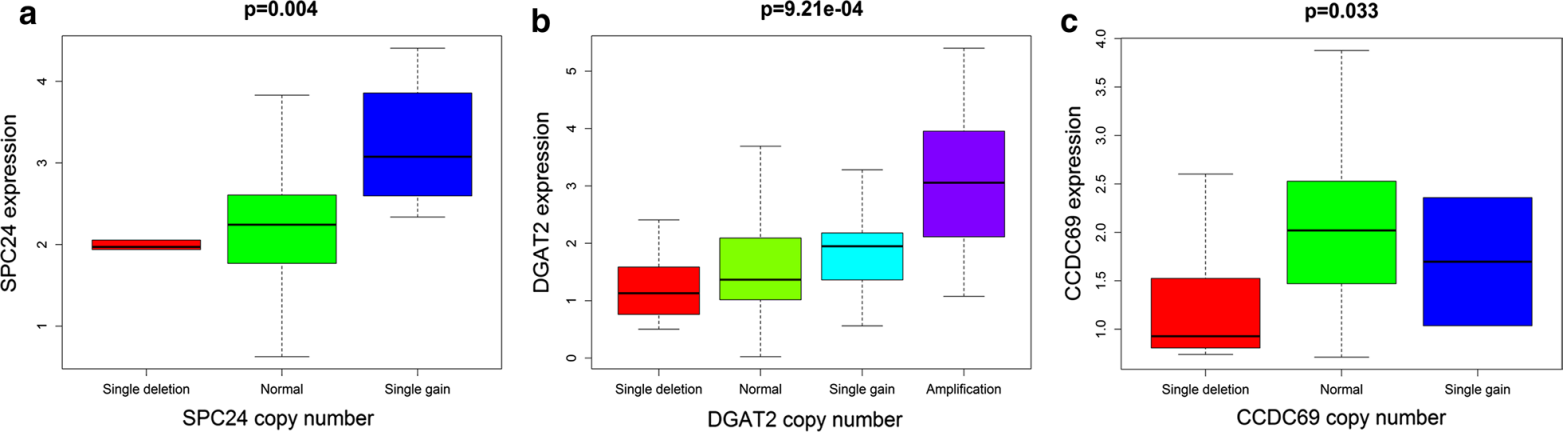
Correlation analysis between CNV mutation and mRNA expression. **a** SPC24; **b** DGAT2; **c** CCDC69

With | Cor | > 0.3 as the cutoff condition, the correlation analysis of 6 mRNAs in the risk scoring system showed that there was a positive correlation between SPC24 and SPC25; a negative correlation between SPC24, SPC25, and CCDC69; and a positive correlation between CCDC69 and DGAT2 (Fig. 7). The results showed that there was some relationship between mRNA and the progression of Her2-positive breast cancer as well as a relationship between mRNAs, which affect each other in the progression of Her2-positive breast cancer. Interestingly, all six mRNAs had the probability of CNV mutation in Her2-positive breast cancer samples, but no CNV mutation was found in normal samples. Among them, RDH16, SPC24, SCUBE3 and DGAT2 were mainly mutations with increased copy number, while CCDC69 and SPC25 were mainly mutations with reduced copy number (Fig. 8). Furthermore, a correlation was found between CNV mutations and the expression of DGAT2, SPC24, and CCDC69 (Fig. 9). Among these, the expression of DGAT2 and SPC24 increased as their copy numbers increased, while an increase or decrease in CCDC69 copy number led to a decrease in its expression. The results showed that there was not only a difference in mRNA expression between normal and Her2-positive samples, but also a difference in the probability of CNV mutation.

## Discussion

Human epidermal growth factor receptor 2 (Her2)-positive breast cancer is a highly dangerous breast cancer subtype, accounting for about 15–20% of breast cancer patients [14, 15]. Although anti-Her2 targeting drugs such as trastuzumab, pertuzumab and ado-trastuzumab emtansine significantly improved the prognosis of patients with Her2-positive early breast cancer, these drugs not only improved the therapeutic effect, but also increased the treatment cost and adverse reactions [16–18]. The exploration of prognostic factors of Her2-positive early breast cancer patients and construction of a risk scoring system for prognostic assessment are of great significance for the screening and diagnosis of patients with high risk of recurrence.

CNV is an important potential source of human phenotypic variation that can affect many fragments in the genome and is closely related to the mechanism of cancer. It has been found that CNV in the GSTM1 gene increases the risk of bladder cancer [19], while Tanenbaum et al. found that CNV mutation in TNFRSF10C is closely associated with distant metastasis in colorectal cancer patients [20]. Thus, it can be seen that tumor CNV research is very important for future targeted therapies for tumor patients. Clinicians can utilize personalized treatment methods according to the difference in copy number in patients to reduce the occurrence of adverse reactions and improve the clinical efficacy.

In order to construct a Her2-positive breast cancer risk scoring system, we screened 761 differentially expressed mRNAs from 183 related samples from a TCGA cohort. Six mRNAs were screened and identified according to univariate and Lasso Cox regression model analyses to construct the prognostic risk scoring system. The expression levels of six mRNAs were correlated with OS, including four up-regulated mRNAs (RDH16, SPC25, SPC24, and SCUBE3) and two down-regulated mRNAs (DGAT2 and CCDC69). The risk scoring system can predict the prognosis of patients with Her2-positive breast cancer by dividing them into high-risk and low-risk groups. In addition, whether it is through a time-dependent ROC curve or a comparison with clinical factors such as age and TNM, the risk scoring system that we constructed has high predictive sensitivity and performance. Finally, based on correlation and CNV mutation analyses, we further explored the research value of the six mRNAs involved in the risk scoring system as the key genes of the model.

Nerve growth factor (NGF) is thought to play an important role in regulating hepatic fibrosis [21] and carcinogenesis [22], while Kaoa et al. found that NGF supplementation significantly increased the expression of retinol dehydrogenase 16 (RDH16). It is suggested that RDH16 may be an important target for the treatment of chronic liver disease [23]. In addition, Hu et al. found that nervous system polycomb-1 (NSPc1) could inhibit the synthesis of all-trans retinoic acid in malignant gliomas by binding to the upstream region of the RDH16 promoter, thus promoting the self-renewal of cancer stem cells [24]. While there are few studies on RDH16 in breast cancer, RDH16 has been shown to mainly affect retinol metabolism to participate indirectly in breast cancer occurrence and progression.

The nuclear division cycle 80 (NDC80) complex is a heterotetrameric protein complex composed of NDC80, Nuf2, SPC24, and SPC25, which plays an important role in mitosis [25]. The components of the NDC80 complex are abnormally expressed in tumors and can be used as biomarkers or prognostic indicators of some tumors. Among these, spindle component 24 (SPC24) could activate the EGFR/Ras/Raf/MEK/ERK signaling pathway and promote tumorigenesis. EGFR and p-ERK expression decreased significantly after Spc24 gene knockout, and osteosarcoma cell growth was significantly inhibited [26]. In addition, Zhou et al. found that SPC24 is significantly overexpressed in breast cancer, which coincides with our results. Furthermore, in both MCF-7 and MDA-MB-231 breast cancer cell lines, it was confirmed that SPC24 knockdown leads to slower cell growth and increased apoptosis [27]. Our study also found that SPC24 had the possibility of CNV mutation in Her2-positive breast cancer, and the expression increased as copy number increased. Because SPC24 is a high-risk factor (i.e., the higher the expression, the greater the risk), this indicates that the CNV mutation of the gene will be indirectly involved in Her2-positive breast cancer progression by affecting its expression level. Some progress has also been made in the study of spindle component 25 (SPC25) in tumor progression. For example, Jeong et al. found that SPC25 expression was up-regulated in a variety of tumor cells [28, 29], which can affect the progression and prognosis of breast cancer patients by regulating the cell cycle, DNA damage repair, and cell proliferation [30, 31].

Signal peptide-CUB-EGF domain-containing protein 3 (SCUBE3) is a secretory cell surface glycoprotein, which is rarely reported in breast cancer but has been overexpressed in a variety of other tumor tissues. It has been found that the fragments released by MMP-2 and MMP-9 cleavage of SCUBE3 can bind to transforming growth factor-b (TGF-b) type II receptors, thus activating TGF-b signal transduction to promote tumor progression [32]. In addition, Chou et al. and Liang et al. found that SCUBE3 was overexpressed in osteosarcoma cells of invasive lung cancer and was closely related to patient prognosis [33, 34]. Our study found that SCUBE3 also showed a trend of overexpression in Her2-positive breast cancer, and the related mechanism of its participation in this progression needs to be further explored.

Diglyceride acyltransferase-2 (DGAT2) and coiled-coil domain-containing protein 69 (CCDC69) are two mRNAs that are lowly expressed in Her2-positive breast cancer and are involved in the risk scoring system. Univariate analysis showed that DGAT2 was a high risk factor, while CCDC69 was a low risk factor. Among them, DGAT2 is also a very important enzyme in organisms, as it plays a crucial role in lipid metabolism. It is reported that DGAT2 expression in HCC tissues is lower than that in normal samples and is positively correlated with the survival time of patients (i.e., the higher the expression level, the longer the survival time); hence, it can be used as a potential anticancer target and diagnostic marker [35]. In addition, it has been found that CCDC69 overexpression can increase the sensitivity of ovarian cancer cells to cisplatin by activating the p14/MDM2/p53 pathway [36], with high expression indicating longer survival time. In our study, the expression of DGAT2 and CCDC69 in Her2-positive breast cancer was decreased and was closely related to patient prognosis; thus, they could be used as biomarkers to evaluate the condition of patients. More surprisingly, both DGAT2 and CCDC69 have CNV mutations in Her2-positive breast cancer; DGAT2 expression increases as copy number increases, whereas an increase or increase in CCDC69 copy number results in a decrease in its expression. According to this result, we can find that the CNV mutations of DGAT2 and CCDC69 can indirectly affect patient prognosis by affecting their expression. Until now, there have been few reports regarding these two kinds of mRNA in breast cancer. The mechanism by which CNV mutation influences expression in breast cancer needs to be further studied.

The TCGA database provides more complete data related to Her2-positive breast cancer, including much data regarding RNA expression, CNV mutation, methylation, and clinical information. In this study, the mRNA risk scoring system developed using the information provided by the TCGA database can effectively predict the prognosis of Her2-positive breast cancer patients. Moreover, both ROC curve verification and univariate and multivariate analyses combined with clinical factors (such as age and TNM) demonstrated that the risk scoring system showed high performance in predicting specificity and sensitivity. The related mRNAs involved in the risk scoring system provide new ideas and directions for further exploration of the mechanism and treatment strategy of Her2-positive breast cancer. However, the current research still has some limitations. Further clinical and experimental verification and more testing methods are needed to improve the accuracy of the prediction model.

## Conclusion

In this study, a risk scoring system based on six kinds of mRNA was constructed that can classify Her2-positive breast cancer patients into different grades according to mRNA expression in order to evaluate patient prognosis. Moreover, some CNV mutations increase patient risk by affecting the expression of the corresponding mRNA, and the presentation of these results contributes to early diagnosis and the implementation of personalized treatment. Although the important molecular mechanisms of these mRNAs in the carcinogenesis of Her2-positive breast cancer need to be further confirmed, the risk scoring system we developed provides important theoretical bioinformatics support for evaluating the prognosis of patients.

## Data Availability

All data generated or analysed during this study are included in this published article.

## Abbreviations

CNV: Gene copy number variation
TCGA: The Cancer Genome Atlas
BP: Biological processes
MF: Molecular function
CC: Cellular component
GO: Gene ontology
KEGG: Kyoto Encyclopedia of Genes and Genomes
OS: Overall survival
LASSO: Least absolute shrinkage and selection operator
ROC: Receiver operating characteristic

## References

[1] Witton CJ, Reeves JR, Going JJ, Cooke TG, Bartlett JM (2003). Expression of the HER1-4 family of receptor tyrosine kinases in breast cancer. J Pathol.

[2] Ross JS, Slodkowska EA, Symmans WF, Pusztai L, Ravdin PM, Hortobagyi GN (2009). The HER-2 receptor and breast cancer: ten years of targeted anti-HER-2 therapy and personalized medicine. Oncologist.

[3] Slamon DJ, Clark GM, Wong SG, Levin WJ, Ullrich A, McGuire WL (1987). Human breast cancer: correlation of relapse and survival with amplification of the HER-2/neu oncogene. Science.

[4] Cumberbatch MGK, Jubber I, Black PC, Esperto F, Figueroa JD, Kamat AM, Kiemeney L, Lotan Y, Pang K, Silverman DT, Znaor A, Catto JWF (2018). Epidemiology of bladder cancer: a systematic review and contemporary update of risk factors in 2018. Eur Urol.

[5] Zhang N, Wang M, Zhang P, Huang T (2016). Classification of cancers based on copy number variation landscapes. Biochem Biophys Acta.

[6] Li BQ, You J, Huang T, Cai YD (2014). Classification of non-small cell lung cancer based on copy number alterations. PLoS ONE.

[7] Pan X, Hu X, Zhang YH, Chen L, Zhu L, Wan S, Huang T, Cai YD (2019). Identification of the copy number variant biomarkers for breast cancer subtypes. Mol Genet Genomics.

[8] Rini B, Goddard A, Knezevic D, Maddala T, Zhou M, Aydin H, Campbell S, Elson P, Koscielny S, Lopatin M (2015). A 16-gene assay to predict recurrence after surgery in localised renal cell carcinoma: development and validation studies. Lancet Oncol.

[9] Peng D, Ge G, Xu Z, Ma Q, Shi Y, Zhou Y, Gong Y, Xiong G, Zhang C, He S (2018). Diagnostic and prognostic biomarkers of common urological cancers based on aberrant DNA methylation. Epigenomics.

[10] Yu SL, Chen HY, Chang GC, Chen CY, Chen HW, Singh S, Cheng CL, Yu CJ, Lee YC, Chen HS (2008). MicroRNA signature predicts survival and relapse in lung cancer. Cancer Cell.

[11] Tomczak K, Czerwińska P, Wiznerowicz M (2015). The Cancer Genome Atlas (TCGA): an immeasurable source of knowledge. Contemp Oncol.

[12] Jiao X, Sherman BT, Da Huang DW, Stephens R, Baseler MW, Lane HC, Lempicki RA (2012). DAVID-WS: a stateful web service to facilitate gene/protein list analysis. Bioinformatics.

[13] Gao J, Kwan PW, Shi D (2010). Sparse kernel learning with LASSO and Bayesian inference algorithm. Neural Netw.

[14] Wolff AC, Hammond ME, Hicks DG, Dowsett M, McShane LM, Allison KH, Allred DC, Bartlett JM, Bilous M, Fitzgibbons P (2013). Recommendations for human epidermal growth factor receptor 2 testing in breast cancer: American Society of Clinical Oncology/College of American Pathologists clinical practice guideline update. J Clin Oncol.

[15] Goutsouliak K, Veeraraghavan J, Sethunath V, De Angelis C, Osborne CK, Rimawi MF, Schiff R (2019). Towards personalized treatment for early stage HER2-positive breast cancer. Nature Rev Clin Oncol.

[16] Pondé N, Amaye L, Lambertini M, Paesmans M, Piccart M, de Azambuja E (2020). Trastuzumab emtansine (T-DM1)-associated cardiotoxicity: pooled analysis in advanced HER2-positive breast cancer. Eur J Cancer.

[17] Swain SM, Baselga J, Kim SB, Ro J, Semiglazov V, Campone M, Ciruelos E, Ferrero JM, Schneeweiss A, Heeson S, Clark E, Ross G, Benyunes MC, Cortés J, CLEOPATRA Study Group (2015). Pertuzumab, trastuzumab, and docetaxel in HER2-positive metastatic breast cancer. N Engl J Med.

[18] Adamczyk A, Kruczak A, Harazin-Lechowska A, Ambicka A, Grela-Wojewoda A, Domagała-Haduch M, Janecka-Widła A, Majchrzyk K, Cichocka A, Ryś J, Niemiec J (2018). HER2Relationship between gene status and selected potential biological features related to trastuzumab resistance and its influence on survival of breast cancer patients undergoing trastuzumab adjuvant treatment. OncoTargets Ther.

[19] Bonberg N, Taeger D, Gawrych K, Johnen G, Banek S, Schwentner C, Sievert KD, Wellhäußer H, Kluckert M, Leng G (2013). Chromosomal instability and bladder cancer: the UroVysion(TM) test in the UroScreen study. BJU Int.

[20] Tanenbaum DG, Hall WA, Colbert LE, Bastien AJ, Brat DJ, Kong J (2016). TNFRSF10C copy number variation is associated with metastatic colorectal cancer. J Gastrointest Oncol.

[21] Kendall TJ, Hennedige S, Aucott RL, Hartland SN, Vernon MA, Benyon RC, Iredale JP (2009). p75 Neurotrophin receptor signaling regulates hepatic myofibroblast proliferation and apoptosis in recovery from rodent liver fibrosis. Hepatology.

[22] Kishibe K, Yamada Y, Ogawa K (2002). Production of nerve growth factor by mouse hepatocellular carcinoma cells and expression of TrkA in tumor-associated arteries in mice. Gastroenterology.

[23] Kao YH, Lee PH, Chiu TC, Lin YC, Sun CK, Chen PH, Tsai MS (2018). Transcriptome analysis reveals a positive role for nerve growth factor in retinol metabolism in primary rat hepatocytes. Cytokine.

[24] Hu PS, Xia QS, Wu F, Li DK, Qi YJ, Hu Y, Wei ZZ, Li SS, Tian NY, Wei QF (2017). NSPc1 promotes cancer stem cell self-renewal by repressing the synthesis of all-trans retinoic acid via targeting RDH16 in malignant glioma. Oncogene.

[25] Suzuki A, Badger BL, Haase J, Ohashi T, Erickson HP, Salmon ED, Bloom K (2016). How the kinetochore couples microtubule force and centromere stretch to move chromosomes. Nat Cell Biol.

[26] Sheng J, Yin M, Sun Z, Kang X, Liu D, Jiang K, Xu J, Zhao F, Guo Q, Zheng W (2017). SPC24 promotes osteosarcoma progression by increasing EGFR/MAPK signaling. Oncotarget.

[27] Zhou J, Pei Y, Chen G, Cao C, Liu J, Ding C, Wang D, Sun L, Xu P, Niu G (2018). SPC24 regulates breast cancer progression by PI3K/AKT signaling. Gene.

[28] Jeong J, Keum S, Kim D, You E, Ko P, Lee J, Kim J, Kim JW, Rhee S (2018). Spindle pole body component 25 homolog expressed by ECM stiffening is required for lung cancer cell proliferation. Biochem Biophys Res Commun.

[29] Cui F, Tang H, Tan J, Hu J (2018). Spindle pole body component 25 regulates stemness of prostate cancer cells. Aging.

[30] Pathania R, Ramachandran S, Mariappan G, Thakur P, Shi H, Choi JH, Manicassamy S, Kolhe R, Prasad PD, Sharma S, Lokeshwar BL, Ganapathy V, Thangaraju M (2016). Combined inhibition of DNMT and HDAC blocks the tumorigenicity of cancer stem-like cells and attenuates mammary tumor growth. Cancer Res.

[31] Wang Q, Zhu Y, Li Z, Bu Q, Sun T, Wang H, Sun H, Cao X (2019). Up-regulation of SPC25 promotes breast cancer. Aging.

[32] Wu YY, Peck KO, Chang YL, Pan SH, Cheng YF, Lin JC, Yang RB, Hong TM, Yang PC (2011). SCUBE3 is an endogenous TGF-β receptor ligand and regulates the epithelial-mesenchymal transition in lung cancer. Oncogene.

[33] Liang W, Yang C, Peng J, Qian Y, Wang Z (2015). The expression of HSPD1, SCUBE3, CXCL14 and Its relations with the prognosis in osteosarcoma. Cell Biochem Biophys.

[34] Chou CH, Cheng YF, Siow TY, Kumar A, Peck K, Chang C (2013). SCUBE3 regulation of early lung cancer angiogenesis and metastatic progression. Clin Exp Metas.

[35] Li Y, Li T, Jin Y, Shen J (2019). Dgat2 reduces hepatocellular carcinoma malignancy via downregulation of cell cycle-related gene expression. Biomed Pharmacother.

[36] Cui L, Zhou F, Chen C, Wang CC (2019). Overexpression of CCDC69 activates p14/MDM2/p53 pathway and confers cisplatin sensitivity. J Ovarian Res.

